# Relation between the Dental Organs and the Uterus

**Published:** 1851-01

**Authors:** W. R. Handy


### ARTICLE II.

*Relation between the Dental Organs and the Uterus.* By Prof. W. R. Handy, M. D.

The fact is well known to all physiologists, and incontestible, that the human body is a unit—that notwithstanding its great variety of parts, all these parts nevertheless act for the common good of the whole, and the whole, in turn, for the separate good of each.

By this mutual relationship, there is consequently established a reciprocal interest, and relative dependency, among the whole and the several parts—and this dependency is so close, that no one part can suffer to any great extent without eliciting in a greater or less degree the sympathy of the whole. And further, the conjoint, though it may be the unperceived actions of the whole for the restoration of the suffering part.

Now this mutual fellow feeling of the whole, and the several parts constituting one common brotherhood, when looked at in its physiological state, presents every thing moving on in such perfect harmony and regularity in the whole body, as well as its different parts, that the attention is seldom called to this dependency, which has just been stated to exist among the different organs. And it is only when this harmony is disturbed and when disease brings discord in this happy family of members, that we find the attention at all roused to the closeness of this dependency which exists among the several organs, and man himself made conscious that he embodies within his own being, a republic of sympathising suffering, and dependent parts—for the suffering member immediately sounds its tale of distress, and the whole family of organs at once take the alarm, and if we be allowed the expression, hold council together, and without waste of time speedily act, and haste to the relief of their suffering brother.

Now the amount of sympathy manifested, or danger threatened as reported by the constitutional disturbance, as a general law, is in proportion to the relative importance of the suffering organ in the general economy. And in compliance with this law the relief sent, or the amount of interest and sympathy manifested on the part of the community of organs, bears also the same proportion. As for instance, it is well known that disease or injury of one of the vital organs, viz. the brain, the heart or lungs, is a much more serious and grave matter than disease or injury to any other organ, for in the first case, we have reason to apprehend the most alarming symptoms, threatening the very citadel of life and its overthrow; while in the latter, as a general rule, we may look to a certain and safe restoration. With this brief statement of general principles, in reference to the dependency of the whole body and its several organs, we come more directly to the subject matter in hand, which is to inquire a little more particularly into the *special relation* of one of these organs with another, viz. that of the *teeth with the uterus*. And the few remarks we have to offer, will be more in the shape of interrogatories, directed to the attention of practical and scientific dentists, as well as physicians, eliciting their personal observations and reflections in the matter, than from any thing that the writer has to offer or can lay claim to, either as settled or conclusive.

In the first place, why, and how is it, that the teeth should sympathise with the womb both in the single as well as the married state? in the absence as well as in the presence of pregnancy? Is such sympathy physiological or pathological? If the former, how does it square with the general law which asserts, that in the state of health all the organs act in such perfect harmony, that the mind takes no cognizance, and is not at all aware, of their several movements? If the latter, that is pathological, why is it not right to interfere and arrest the diseased action? And further, whether this state of sympathy be healthy or diseased, are there any observations either past or present of a practical nature, likely to result in a more improved practice for the comfort and benefit of the female?

In reference to the first query, it may be stated that as to the why and how of the sympathy of the teeth with the uterus, that the answer is as readily given, and the explanation as easily understood, as in the familiar cases of trismus and hydrophobia. We do not think the cases exactly analogous, for in that of trismus, the locked-jaw is most generally the result of injury, while hydrophobia is caused by the introduction of poison into the system, both affections arising from a pathological state, peculiar and inexplicable it may be, but is, nevertheless, pathological and must certainly consist in such an altered condition from the natural healthy character of the injured part or organ, as to produce the peculiar symptoms belonging to each affection. But in the case of the teeth and uterus there seems to be no disease in either the one or the other. It is true the female may have the most violent tooth-ache, yet, on examination, the dentist pronounces nothing to be the matter with the teeth and that they are all perfectly sound. And for the uterus no complaint has been made in reference to it, and the general health seems in every respect to be good. In the unmarried female, this pain of the teeth seems to be present and most especially connected with the period of menstruation, while in the married it is felt most during pregnancy, and that this peculiar tooth-ache is associated with the uterus in preference to every other organ, seems manifest from the fact, that there is no such complaint either before menstruation has appeared or after it has ceased.

Now, it is admitted by all physiologists that both menstruation and pregnancy are healthy actions, and if so, how should they produce an unhealthy action, such as pain in any part? and more especially in a part so distant as the teeth? If it be denied that the menstruation and pregnancy (when this pain of the teeth is complained of) are entirely healthy, the question then recurs, viz. what is the pathological condition? Is it a change of function or structure in the uterus? are the nerves or blood vessels diseased? and if so, what is the character of their derangement? or is it the molecular or nutrition of the womb that is affected? and if one or more of these diseased conditions should be present, what special relation have they with the teeth, that the latter should sympathise with, and report the existence of such a state of things in preference to the mama or any of the group of organs so intimately connected with the uterus and comprising the genital system? Now it is well known that all diseases of the uterus do not give pain in the teeth, nor that every pain of a tooth by any means depends on disease of the womb. Well, how are we to diagnose the character of the pain? or is there any method or utility in making any discrimination?

Acid fluids of the mouth, or disordered digestion, as well as disease of the uterus, may all give rise to pain of the teeth. And in the two first cases, a treatment would be recommended and applied, not to the teeth themselves, but to the removal of the causes on which the pain depends; in the one case to the correction of the acrid qualities of the fluids of the mouth, and in the other, to the restoration of the disordered digestion of the stomach. If treatment be acknowledged as necessary in these two cases to remove this painful sympathy of the teeth, why is it not equally necessary when the uterus is the cause, and command an equal amount of attention both on the part of the patient and physician? Is it because there is greater difficulty in detecting disorders of the uterus, and tracing their effects upon other and distant organs? or is it because there has been less attention given to this species of relationship, and that cause and effect between the teeth and the uterus have not been so industriously looked after, or so carefully watched, as in other cases of sympathetic action? This latter reason we suspect to be the true one, and if so, the course to be pursued by every medical man and scientific dentist seems to be, to give closer observation into the various sympathies of the uterus with distant organs; and endeavor to ascertain what the precise change from the healthy condition of the uterus consists in. Whether this change refers to an alteration in the structure or function, or both of these organs. In other words, what constitutes the exact pathological state, if it be such, of the uterus upon which those very peculiar and remote symptoms depend? If such discrimination be possible, we shall then be furnished with an intelligible data upon which to predicate a rational practice—a practice so reliable that we will know whether it is best to do nothing, or whether, on the contrary, it is best to promptly interfere. One word more, in reference to the first query, of "why and how" the uterus should sympathise with the teeth? and we close these rather desultory remarks. When the question is asked, why and how is it, that an organ sympathises with some one in preference to another? we look for the answer, and reply, that it is owing either to its contiguity, or is concerned in the performance of the same function, and hence indicating the closest fellowship and most intimate relation. But there may be neither this contiguity nor especial participation in function, and yet a very close connection by nerves, as we see between the brain and the stomach, by means of the par vagum, which passes directly from the one organ to the other; and so close is this connection, that injury to the brain, or distressing news, it is well known, not only may, but frequently does, produce loathing of the food and vomiting; and *vice versa*, disorder of the stomach is reflected to and disturbs the brain.

But in the case of the teeth and the uterus, there is no such relation between them, either by contiguity, by direct intercourse through nerves, or by any special connection in the performance of function; yet it is manifest there must be a relationship, through some channel or other, to explain the presence of symptoms, which clearly flow as cause and effect, and as clearly point to the dependency which evidently exists between these two sets of organs.

Then the question is, what is this channel of intercourse? A man has locked-jaw from injury of the toe, a sympathy even more remote, and quite as obscure as that of the teeth and the uterus, and no one questions this relationship—but how does it exist? Now, our own opinion is, that the medium of connection is the same in both cases, and that the sentient tract of the spinal marrow is this medium. In the one case, the sentient nerves of the uterus, being impressed by the changes occurring in this organ at the periods of menstruation and pregnancy, reflect this impression to the sentient tract of the spinal marrow, whence it is conducted upwards, along this tract, to the point where the fifth pair of nerves pass off; and here it takes the route of this nerve—the great sentient nerve of the teeth and the face—going along the dental branches where there is pain in the teeth, and, by a reflex action, to the muscles of the lower jaw, in the case of *trismus*, where the impression from the sentient nerves of the toe are also reflected to the spinal marrow.

